# Current Status on the Functional Characterization of Chemosensory Receptors of *Cydia pomonella* (Lepidoptera: Tortricidae)

**DOI:** 10.3389/fnbeh.2018.00189

**Published:** 2018-08-27

**Authors:** Alberto Maria Cattaneo

**Affiliations:** Division of Chemical Ecology, Department of Plant Protection Biology, Swedish University of Agricultural Sciences, Alnarp, Sweden

**Keywords:** *Cydia pomonella*, chemosensory receptors, functional characterization, *Drosophila* empty neuron system, human embryonic kidney (HEK293T) cells

## Abstract

*Cydia pomonella* (Lepidoptera: Tortricidae) is a major pest of apple, pear and walnuts. For its control, alternative strategies targeting the olfactory system, like mating disruption, have been combined with insecticide applications. The efficacy of these strategies headed the direction of efforts for the functional characterization of codling moth chemosensory receptors to implement further control methods based on chemical sensing. With the advent of transcriptomic analysis, partial and full-length coding sequences of chemosensory receptors have been identified in antennal transcriptomes of *C. pomonella*. Extension of partial coding sequences to full-length by polymerase chain reaction (PCR)-based techniques and heterologous expression in empty neurons of *Drosophila melanogaster* and in Human Embryonic Kidney cells allowed functional studies to investigate receptor activation and ligand binding modalities (deorphanization). Among different classes of antennal receptors, several odorant receptors of *C. pomonella* (CpomORs) have been characterized as binding kairomones (CpomOR3), pheromones (CpomOR6a) and compounds emitted by non-host plants (CpomOR19). Physiological and pharmacological studies of these receptors demonstrated their ionotropic properties, by forming functional channels with the co-receptor subunit of CpomOrco. Further investigations reported a novel insect transient receptor potential (TRPA5) expressed in antennae and other body parts of *C. pomonella* as a complex pattern of ribonucleic acid (RNA) splice-forms, with a possible involvement in sensing chemical stimuli and temperature. Investigation on chemosensory mechanisms in the codling moth has practical outcomes for the development of control strategies and it inspired novel trends to control this pest by integrating alternative methods to interfere with insect chemosensory communication.

## Introduction

The codling moth *Cydia pomonella* (Lepidoptera: Trotricidae) is a major pest insect of commercial crops such as apple, pear and walnuts of Palearctic and Nearctic regions (Witzgall et al., 2008).

Integrated with insecticides, alternative methods are commonly used to control this insect (Starà et al., 2008; Odendaal et al., 2015; Arnault et al., 2016; Iraqui and Hmimina, 2016). Among these methods, mating disruption, which targets the olfactory system of *C. pomonella* males through the use of female sex pheromones, demonstrated efficient results to limit crop infestation (Hathaway et al., 1974; Ridgway et al., 1990; Light et al., 2001; Light, 2016). Furthermore, odors emitted by host-plants (kairomones), are combined with pheromones to enhance male attraction for the codling moth (Knight and Light, 2001; Light et al., 2001; Witzgall et al., 2001, 2005; Yang et al., 2004).

In insects, odors such as pheromones and kairomones are detected by olfactory sensory neurons (OSNs) that innervate specialized sensilla on their antennae (Buck and Axel, 1991; Chess et al., 1992; Vosshall et al., 2000; Carlson, 2001; Kurtovic et al., 2007). On the dendritic membrane of OSNs, odorants and pheromones mostly bind a class of seven-transmembrane proteins known as odorant receptors (ORs; Clyne et al., 1999). Deciphering mechanisms of receptor/ligand interactions and understanding pharmacological, kinetic properties and activation modalities of OR proteins, unveil promising aspects to improve strategies for the control of pest insects (Jones et al., 2011; Pask et al., 2011, 2013; Röllecke et al., 2013; Bobkov et al., 2014). Identification of ligands for specific ORs (deorphanization) among odors emitted from females and plant-hosts of the codling moth facilitates our understanding of the neurobiological and behavioral aspects at the base of the chemical ecology of *C. pomonella*. This contributes to possible application of novel ligands for semiochemical-based control strategies.

This mini-review reports the state of the art of current findings on the functional characterization of codling moth ORs as well as findings of a novel transient receptor potential (TRP) channel expressed in the olfactory system of *C. pomonella*. This contribution introduces ongoing studies on the molecular aspects of chemical sensing of the codling moth and their possible application to current control strategies targeting the olfactory system of the insect.

## Identification of Chemosensory Receptors of *Cydia pomonella*

By means of a polymerase chain reaction (PCR)-based technique, the 3’ end of gene transcripts encoding putative members of *C. pomonella* ORs (*CpomORs*) have been initially identified from total ribonucleic acid (RNA) samples extracted from antennae (Garczynski et al., 2012). In this study, a similar method described by Buck and Axel (1991) was used to design degenerate forward primers based on polypeptide sequence alignments of the C-terminus of 12 members of the pheromone receptor subfamily of *Bombyx mori* (Lepidoptera: Bombycidae) and *Heliothis virescens* (Lepidoptera: Noctuidae). Forward primers were used to amplify partial 3’-ends starting from retro-transcribed 3’-cDNA templates generated by SMART™ kits (Clontech, Mountain View, CA, USA). Among amplified 3’-ends, the first set of *CpomORs* were identified. This method represented the first effort in the isolation of *CpomORs* from antennal RNA samples, leading optimization of further RACE-PCR approaches to amplify the full-length coding sequences of other chemosensory genes of this insect, aimed to address their phylogenetic and functional characterization.

With the advent of transcriptomic analysis, a wider investigation was conducted by the use of 454-next generation sequencings (NGS) of antennal RNA-samples (Bengtsson et al., 2012). For the first time, a wide asset of assembled fragments of gene coding sequences was identified, revealing 14 candidate ionotropic receptors (IRs), one candidate gustatory receptor (GR) and 43 candidate ORs. Among these, five ORs were members of the putative pheromone receptors (PRs) subfamily: a monophyletic clade in Lepidopteran insect OR phylogeny, with receptors that predominantly respond to odors emitted by females (Jacquin-Joly and Merlin, 2004; Ihara et al., 2013; Leal, 2013). Among the five candidate PRs reported by Bengtsson et al. (2012), two PRs represented some of the same ORs identified in the previous work of Garczynski et al. (2012). With the aim to complement these studies, using Illumina-based RNA-sequencing, assembly of a transcriptome from male, female and larval olfactory tissues of the codling moth, a more complete list of chemosensory receptors of *C. pomonella* was updated to 21 IRs, 20 GRs and 58 putative ORs, among which, 11 represented members of the PR-clade (Walker et al., 2016; Table 1). Identification of IRs and GRs in antennal transcriptomes of the codling moth was in accordance with the reported findings of their functional importance in insect chemosensation (Clyne et al., 2000; Robertson et al., 2003; Benton et al., 2009; Montell, 2009; Ai et al., 2010; Silbering et al., 2011; Rytz et al., 2013; Missbach et al., 2014; Sanchez-Alcaniz et al., 2018). Despite their importance, most of the efforts to functionally characterize chemosensory receptors of the codling moth targeted ORs, with particular focus on members of the PR-subfamily.

**Table 1:** Updated list of *Cydia pomonella* odorant receptors (CpomORs) in comparison among results from Garczynski et al. (2012); Bengtsson et al. (2012) and Walker et al. (2016), based on their techniques (brackets).

Walker et al. (2016)	Bengtsson et al. (2012)	Garczynski et al. (2012)	Status	Clade
(*Illumina*)	(*454*)	(*RACE-PCR*)		
CpomOrco	CpomOR2	-	Complete^a^	Co-receptor
CpomOR1	CpomOR4	CpomOR11	Complete^a,b^	PR^M^
CpomOR2a	CpomOR5	CpomOR1a	Incomplete^b^	PR
CpomOR2b		CpomOR1a
CpomOR2c		CpomOR11a
CpomOR3	CpomOR3	-	**Deorphanized^a,b;1,2^**	PR
CpomOR4	CpomOR6	CpomOR4	Complete	PR
CpomOR5	-	-	Complete^b^	PR^M^
CpomOR6a	CpomOR1	-	**Deorphanized^a,b;2^**	PR^M^
CpomOR6b	-		Complete^b^
CpomOR7	-	-	Complete	PR^M^
CpomOR8	-	-	Complete	PR
CpomOR9	-	-	Incomplete	PR
CpomOR10	CpomOR28	-	Complete^b^	OR
CpomOR11	CpomOR11	-	Incomplete	OR^L^
CpomOR12	-	-	Incomplete	OR
CpomOR13	CpomOR8	-	Complete	OR
CpomOR14	CpomOR14	-	Complete	OR
CpomOR15	CpomOR20	-	Complete	OR
CpomOR16	-	-	Complete	OR
CpomOR18	CpomOR10	-	Complete	OR^L^
CpomOR19	CpomOR19	-	**Deorphanized^b;3^**	OR
CpomOR20	CpomOR18	-	Complete	OR
CpomOR21	-	-	Incomplete	PR^F^
CpomOR22	CpomOR15	-	Complete^b^	PR^F^
CpomOR25	CpomOR21	-	Complete	OR
CpomOR26	-	-	Complete	OR
CpomOR27	CpomOR27	-	Complete	OR
	CpomOR29
CpomOR28	CpomOR26	-	Complete^b^	OR
CpomOR29	-	-	Complete	OR
CpomOR30	CpomOR30	-	Complete	OR^F^
CpomOR31	-	-	Complete	OR^M^
CpomOR32	-	-	Complete	OR
CpomOR35	CpomOR35	-	Complete	OR
CpomOR37	CpomOR36	-	Complete	OR
	CpomOR39
CpomOR38	-	-	Incomplete	OR
CpomOR39	CpomOR38	-	Complete^b^	OR
CpomOR40	CpomOR33	-	Complete	OR
CpomOR41	-	-	Complete	OR^F^
CpomOR42	-	-	Complete	OR
CpomOR44	-	-	Complete	OR
CpomOR46	CpomOR16	-	Complete	OR
CpomOR47	-	-	Complete	OR
CpomOR49	-	-	Complete	OR
CpomOR53	CpomOR9	-	Complete	OR
CpomOR54	CpomOR7	-	Complete	OR
	CpomOR41			
CpomOR56	CpomOR37	-	Complete	OR
CpomOR57	CpomOR31	-	Complete	OR
CpomOR58	CpomOR34	-	Complete	OR
CpomOR59	CpomOR12	-	Complete	OR
CpomOR60	-	-	Complete	OR
CpomOR61	CpomOR17	-	Complete	OR
CpomOR62	-	-	Complete	OR
CpomOR63	CpomOR23	-	Complete	OR
CpomOR64	CpomOR24	-	Complete	OR^L^
CpomOR65	CpomOR22	-	Complete	OR
CpomOR66	CpomOR32	-	Incomplete	OR
CpomOR67	-	-	Complete	OR
CpomOR68	-	-	Complete	OR
CpomOR71	-	-	Complete^b^	OR^L^
CpomOR72	CpomOR40	-	Complete	OR
Not found	CpomOR13	-	-	-
Not coding	CpomOR43	-	-	-
Not coding	CpomOR44	-	-	-

**Figure 1:**
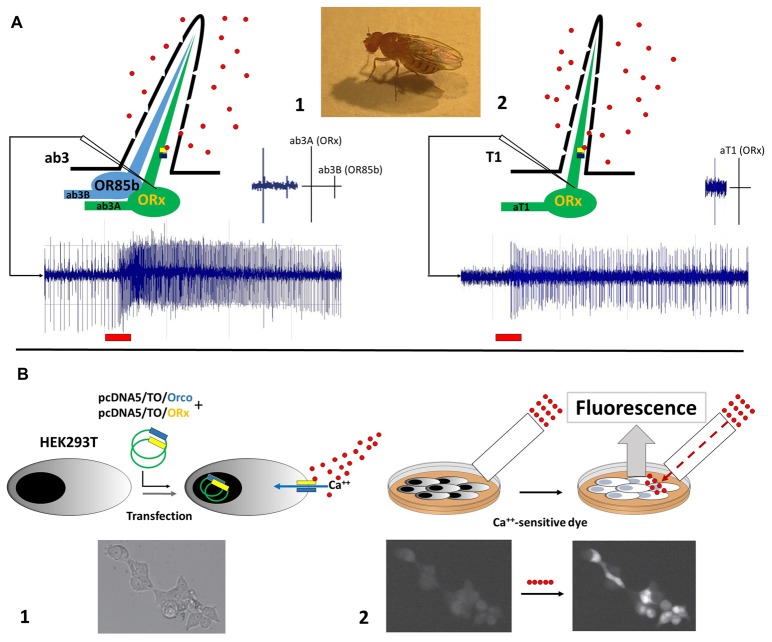
Illustration of heterologous expression methods adopted to functionally characterize odorant receptors (ORs) of *Cydia pomonella*. **(A)** Functional expression in empty neurons of *D. melanogaster*, between ab3A (1) and aT1 (2) neurons. Spike trains example are provided below; red bars indicate stimulation period. Spike scales between ab3A/B and aT1 are shown on the top-right. Recording electrodes are shown as white triangles. **(B)** Functional expression in Human Embryonic Kidney (HEK293T) cells: (1) transfection of HEK293T cells with plasmids carrying coding sequences of Orco (blue) and ORx (yellow): activation of the Orco+ORx cation channel by ligand-binding is represented; calcium-influx in response to Orco+ORx activation is indicated with a blue arrow. (2) Incubation of HEK cells with a Ca^++^-sensitive dye: fluorescent response elicited by calcium-influx through the cation channel is represented. Perfusion system is shown as a white rectangle. Ligands are represented as red circles. Orco+ORx is represented as blue/yellow bars. Images of HEK293T cells at the bottom are part of our published data (Cattaneo et al., 2017b). Licence to the use of the material reported in the aforementioned publication is available at the following link: http://creativecommons.org/licenses/by/4.0/.

*^M^Receptors with male-bias expression; ^F^receptors with female-bias expression; ^L^receptors with larval-bias expression (Walker et al., 2016). ^a^Receptors heterologously expressed in Human Embryonic Kidney (HEK293T); ^b^receptors heterologously expressed in *Drosophila* empty neurons (Figure 1). Deorphanized receptors are indicated with numbers based on published data: ^1^Bengtsson et al. (2014); ^2^Cattaneo et al. (2017b); ^3^Gonzalez et al. (2015). Accession to the updated dataset of CpomORs is available in Walker et al. (2016)*.

## Functional Characterization of CpomOR3

CpomOR3 represents the first OR of the codling moth that was isolated, heterologously expressed and functionally characterized (Bengtsson et al., 2014). Expression of this receptor was conducted in *Drosophila melanogaster* ab3A (Dobritsa et al., 2003; Gonzalez et al., 2016) and aT1 (Kurtovic et al., 2007; Montagné et al., 2012) empty neurons, to screen a panel of ligands among pheromones, synergists and antagonists known for their activation of the olfactory system of *C. pomonella*. Activation of CpomOR3 was demonstrated for the plant volatile ethyl-E,Z-2,4-decadienoate, commonly known as pear ester (Jennings et al., 1964; Berger and Drawert, 1984; Light et al., 2001; Yang et al., 2004; Knight et al., 2005; Willner et al., 2013). Phylogenetic analysis demonstrated CpomOR3 to be a PR-candidate. Activation of a putative PR to the synergist pear ester was in accordance with neurological effects identified when this compound was tested with the primary sex pheromone component of the codling moth (codlemone) on AL-glomeruli of the insect (Trona et al., 2010, 2013). CpomOR3 response to pear ester gave further support to the role of this compound as a kairomone, already known to enhance male attraction in orchards when combined with female pheromones (Light et al., 2001; Yang et al., 2004). These findings suggest a possible role of kairomones in the mate-choice behavior and in the reproductive isolation of tortricids (Trona et al., 2013; Bengtsson et al., 2014).

To better elucidate mechanisms involving pear ester sensing for CpomOR3, functional characterization experiments based on *Drosophila* empty neurons have been implemented through the heterologous expression of this receptor in Human Embryonic Kidney (HEK293T) cells (Cattaneo et al., 2017b). The use of an *in vitro* method represented an alternative to the common approaches for the functional characterization of insect ORs based on *Drosophila* empty neurons. The choice of this method was supported by successful attempts on the expression of PR-candidates from moths belonging to Bombycidae (Grosse-Wilde et al., 2006), Noctuidae (Grosse-Wilde et al., 2007), Saturniidae (Forstner et al., 2009) and Tortricidae (Steinwender et al., 2015). Comparison of heterologous expression between *Drosophila* OSNs and HEK293T is shown in Figure 1.

In search of other possible ligands for CpomOR3, screening of a compound library on HEK293T cells validated activation of the receptor to both pear ester and the analogous methyl-(E, Z)-2, 4-decadienoate. Sensing of an analogous methyl-ester for the codling moth was reported for the first time by demonstrating larval attraction from emissions of ripe Bartlett pear (Knight and Light, 2001), although origins of methyl ester as a plant-emitted odorant are still debated. Indeed, aside from emission by Bartlett pear, methyl ester was found in the head, thoraxes and fecal pellets of the bark beetle *Pityogenes chalcographus* (Coleoptera: Curculionidae; Birgersson et al., 1990). In addition, methyl ester was also found in emissions from stink bugs of the genus *Euschistus* (Heteroptera: Pentatomidae; Aldrich et al., 1991; Tognon et al., 2016).

A remaining question is if interaction of the analogous methyl ester with the same receptor of ethyl-(E,Z)-2,4-decadienoate may result in a similar effect in the antennal lobe as an evidence of its synergism with codlemone (Trona et al., 2010, 2013).

## Functional Characterization of CpomOR6a

Heterologous expression of codling moth receptors in HEK293T cells also deorphanized the PR candidate CpomOR6a as responsive to (E, E)-8–12-dodecadien-1-yl acetate (Codelmone acetate; Cattaneo et al., 2017b). Combining heterologous expression in *Drosophila* aT1, (E,Z)- and (Z,Z)-geometric isomers of codlemone acetate were also identified as partial ligands of the receptor. Together with these ligands, CpomOR6a sensed (E)-10-dodecadien-1-yl acetate and, although with less specificity, (Z,E)-8–12-dodecadien-1-yl acetate.

Codlemone acetates are main pheromone components emitted by female moths closely related to *C. pomonella* (Frerot et al., 1979; Roelofs and Brown, 1982; Davis et al., 1984; Witzgall et al., 1996; Chambers et al., 2011). Although receptors of codlemone acetates of these species have not been isolated and deorphanized yet, overall sequence similarities and relatively high expression in *C. nigricana* and *Hedya nubiferana*, suggested the gene locus *OR6* to express a conserved receptor between *C. pomonella* and these tortricid species (Gonzalez et al., 2017). While speculative, a possible explanation of the existence of the codlemone acetate receptor in *C. pomonella* may be as a remnant of the former ancestor of the insect. However, conserving a receptor dedicated to detect other species may be important for reproductive isolation of the codling moth. Otherwise, since moths emitting codlemone acetates share the same host range with *C. pomonella*, detection of codlemone acetates may facilitate host finding for the codling moth. The evolution of a receptor specialized for the detection of a main pheromone compound, like codlemone, may likely represent a step towards allopatric speciation of *C. pomonella*.

Among candidate PRs of the codling moth (Table 1), the most likely sensor for codlemone is CpomOR1 given its abundant expression in OSNs of male moths (Bengtsson et al., 2014; Walker et al., 2016). Although heterologous expression methods in HEK cells and *Drosophila* empty neurons were unable to demonstrate CpomOR1 responsiveness to codlemone (Cattaneo et al., 2017b), future deorphanization attempts will unveil if this prediction holds true. Another remaining question is whether the transcript variant CpomOR6b has the same response spectrum as CpomOR6a and what might be its relevance, especially considering the lack of knowledge on alternative splicing in lepidopteran PRs (Garczynski and Leal, 2015).

## Functional Characterization of CpomOR19

Although CpomOR19 is not a PR-candidate in *C. pomonella*, testing heterologous expression of this receptor in *Drosophila* ab3As is part of documented deorphanizations of chemosensory receptors of the codling moth (Gonzalez et al., 2015).

CpomOR19 is responsive to 1-indanone; several analogs of this compound (2-methyl-1-indanone, 2-ethyl-1-indanone and 3-methyl-1-indanone) elicit responses of different sensitivities by this receptor. These compounds are renowned for their “non-host” origins (Klein et al., 1990; Anderson et al., 1993; Nagle et al., 2000; Okpekon et al., 2009; Rukachaisirikul et al., 2013). CpomOR19 binding to indanes represented the first deorphanization of a receptor of the codling moth to compounds emitted by non-hosts. Interestingly, different sensitivities between 1-indanone and its analogs for the CpomOR19 binding is consistent with observations reported between pear and methy esters on CpomOR3, where one carbon of the alkyl group may determine different binding affinity, perhaps due to differences in the polarity of the compounds (Cattaneo et al., 2017b).

By use of the same method, activation of the ortholog SlitOR19 of the African cotton-leaf worm *Spodoptera littoralis* (Lepidoptera: Noctuidae) demonstrated conservation in binding 1-indanone and analogous. When compared, ab3A expressing CpomOR19 and SlitOR19 showed increased response to indanes, when substituted with alkyl groups at position two and three of the five-membered ring. On the contrary, indanes provided with methyl substituents on the benzene ring largely did not activate these receptors. Furthermore, indanes provided with alcohols, hydrocarbons and amine groups also did not activate any of the two receptors, which suggested a conserved function for CpomOR19 and SlitOR19 orthologs, despite the phylogenetical and ecological distance of their respective moths. A recent report on *Spodoptera* ORs provides a blueprint for prediction of SlitOR ligands based on the interaction of phylogeny and chemical structure (de Fouchier et al., 2017). Given evidences of conserved function between CpomOR19 and SlitOR19, prediction of SlitOR ligands may benefit future studies on deorphanization of CpomORs.

## Physiological Properties of CpomORs

Expression of CpomOR genes in HEK cells was undertaken by co-transfecting the CpomOrco co-receptor subunit of the codling moth (Figure 1B). Functional studies of CpomOrco demonstrated heteromeric complexes of the co-receptor with OR subunits being more sensitive than homomeric co-receptor complexes, as previously demonstrated for Orco-based channels of other insects (Jones et al., 2011; Pask et al., 2011; Kumar et al., 2013; Turner et al., 2014). By the use of the main ligand VUAA (Jones et al., 2011), calcium response was characterized by faster activation/deactivation kinetics for CpomORco+OR than CpomORco alone. Testing inhibitors like amiloride derivatives (ADs; Pask et al., 2013; Röllecke et al., 2013) demonstrated similar effects for both homomeric and heteromeric complexes. When HEK cells were tested by whole-cell and outside-out patch-clamp recordings, activation of CpomOrco+OR complexes resembled modalities of ligand-gated cation channels: responses to multiple stimulations were characterized by constant amplitudes and stable kinetic parameters, which is indicative of the ionotropic nature of insect OR receptors (Sato et al., 2008).

Despite that the molecular mechanisms at the base of signal transduction of insect olfactory systems still remain unknown (Krieger and Breer, 1999; Jacquin-Joly and Merlin, 2004; Sakurai et al., 2014), results on the functional characterization of *C. pomonella* ORs are consistent with the idea that all insect or, perhaps, even all arthropod chemosensory receptor channels (among ORs and IRs) can be characterized by somewhat common pharmacology (Bobkov and Ache, 2007; Abuin et al., 2011; Bobkov et al., 2014). Although, this might be called into question given some evidence pointing towards metabotropic signaling modalities for insect ORs (Sargsyan et al., 2011; Getahun et al., 2013; Ignatious Raja et al., 2014).

## Transient Receptor Potential Channels of *Cydia pomonella*

A second analysis of sequencing data from Bengtsson et al. (2012), unveiled further transcripts related with ligand-gated cation channels belonging to the class of TRP. In several organisms, TRP-channels enable sensing of multiple stimuli from the environment (Liedtke and Heller, 2007). Among chemical stimuli, several compounds commonly found in food plants and spices are reported to activate TRPs (Caterina et al., 1997; Jordt et al., 2004; Xu et al., 2006; Bautista et al., 2007). Interestingly, TRP-active compounds are reported for their ability to repel insects (Leung and Foster, 1996; Barnard, 1999) and, in particular, to activate the olfactory system of tortricid and noctuid moths (Cattaneo et al., 2014; Wei et al., 2015).

In *C. pomonella*, five TRPs have been found in the antennae belonging to the TRPC (TRP, TRPC) and the TRPA subfamily (Pyrexia, water witch, TRPA5; Cattaneo et al., 2016). Up to now, *CpomTRPA5* is the only TRP of the codling moth that has been extended to the full length. Interestingly, five variants of the spliced-coding sequence have been found, demonstrating different expression patterns among body parts of the codling moth. Analysis of the *CpomTRPA5* mRNA sequence demonstrated the transcript undergoing to mRNA editing by insertion of 15 additional nucleotides within the third exon of the full-length sequence, which is a mechanism occurring for K^+^ channels of multiple organisms, including insects (Holmgren and Rosenthal, 2015). Evolutionary studies suggested the relatedness of *TRPA5* gene to the thermal sensor *Pyrexia* (Peng et al., 2015), which has also been descripted as a thermal-gated K^+^-channel of insect (Lee et al., 2005).

Identification of TRPs in *C. pomonella* represented the first documented finding within this species for this particular class of chemoreceptors. Identification of *CpomTRPA5* and its spliceforms is among the first documented existences of this particular subunit for arthropod TRPAs (Peng et al., 2015). Relatedness of *CpomTRPA5* with *Pyrexia* suggested a possible role of the CpomTRPA5 receptor as a thermal sensor, which is consistent with behavioral evidences for the codling moth of odor-guided responses in relation with temperature (Witzgall et al., 1999).

By means of methods adopted to test activation of mammalian TRPAs expressed in HEK cells (Bassoli et al., 2009, 2013; Cattaneo et al., 2017a), functional characterization studies of CpomTRPA5 may be conducted to better elucidate possible roles of this receptor in chemical and physical sensing modalities of the codling moth.

## Future Perspectives

The technologies adapted to the setup of transcriptomic and heterologous expression studies for the functional characterization of chemosensory receptors of *C. pomonella*, may offer new opportunities to address longstanding questions in the field of insect ecology, with a practical outcome for the implementation of its control strategies.

Two out of the three codling moth ORs that have been deorphanized, belong to the clade of putative Pheromone Receptors. Although attempted, the receptor for the main pheromone codlemone has not been functionally characterized. To validate a possible role of CpomOR1 as a main candidate sensor (Bengtsson et al., 2012; Walker et al., 2016; Cattaneo et al., 2017b), future experiments will verify if co-expression of CpomOR1 with CpomOR6a in *Drosophila* aT1 neurons is sensitive to codlemone. This approach is supported by evidences of response to codlemone acetates from OSNs of *C. pomonella* responding to codlemone (Bäckman et al., 2000), which may suggest a possible role of the CpomOR6a subunit to sense this pheromone. In addition, studies on several insects demonstrated co-expression of different OR subunits in the same OSN (Couto et al., 2005; Fishilevich and Vosshall, 2005; Goldman et al., 2005; Ray et al., 2007; Koutroumpa et al., 2014; Karner et al., 2015; Lebreton et al., 2017), and stoichiometry of OR heteromers is still debated (Larsson et al., 2004; Benton et al., 2006; Wicher, 2018).

In support of the control of the codling moth with mating disruption, novel trends are leading the direction of studies to integrate targeting of sensing modalities of codling moth females. Indeed, methods based on mating disruption demonstrated inefficacy to the control of the codling moth at high population in the orchards, as well as on the top of tree branches, where the pheromone cloud is limited (Witzgall et al., 1999). Identification of CpomORs with a female-biased expression (Bengtsson et al., 2012; Walker et al., 2016) motivates the use of heterologous methods to address their functional characterization (Swedish Research Council Formas, Project Reg. No. 2016-01281 “*Control of Apple Pest Insects with Fruit and Yeast Odorants*”). This approach may identify novel ligands active on female olfactory systems. Among these ligands, odors emitted by fruits and their associated microbes may be tested, given the importance of yeasts for attractiveness of egg-lying females (Witzgall et al., 2012).

Recent studies based on CRISPR/Cas9 editing of the codling moth, demonstrated efficacy of this method to address knockdown of functional OR proteins, which resulted in affection of fecundity and fertility, with edited females producing nonviable eggs (Garczynski et al., 2017). Future targets may combine heterologous expression methods, with the use of CRISPR/Cas9 to generate OR-edited insects, as a complementary approach to address the functional characterization of codling moth receptors.

Future trends integrating research on the olfactory system of *C. pomonella* may target larval chemical sensing as complementary to the current approaches addressing the functional characterization of adult ORs (Formas Mobility Starting Grant Reg. No. 2018-00891 “*Control of Fruit Pests by Targeting Larval Chemical Sensing*,” submitted). Indeed, chemosensory mechanisms at the base of larval behavior are long renowned for the codling moth (Knight and Light, 2001; Jumean et al., 2005) and expression of CpomORs with a larval-bias has been reported (Walker et al., 2016).

Broader discoveries on the molecular bases of the olfactory mechanisms of *C. pomonella* will enhance current control strategies interfering with the insect’s chemosensory communication. Development of novel methods targeting olfaction may help limit the use of insecticides, with beneficial effects on the quality of life for apple growers, consumers, as well as public living around the orchard areas, reducing further the conflict between agricultural and urban worlds.

## Author Contributions

AC wrote the manuscript.

## Conflict of Interest Statement

The author declares that the research was conducted in the absence of any commercial or financial relationships that could be construed as a potential conflict of interest.
